# Associations of Cardiac Ventricular Repolarization with Serum Adhesion Molecules and Cognitive Function in Older Adults: The MIND-China Study

**DOI:** 10.3233/JAD-220874

**Published:** 2023-03-07

**Authors:** Chaoqun Wang, Ming Mao, Xiaolei Han, Tingting Hou, Xiaojie Wang, Qi Han, Yi Dong, Rui Liu, Lin Cong, Cuicui Liu, Yume Imahori, Davide L. Vetrano, Yongxiang Wang, Yifeng Du, Chengxuan Qiu

**Affiliations:** a Department of Neurology, Shandong Provincial Hospital, Cheeloo College of Medicine, Shandong University, Jinan, Shandong, P.R. China; b Department of Neurology, Shandong Provincial Hospital affiliated to Shandong First Medical University, Jinan, Shandong, P.R. China; c Shandong Provincial Clinical Research Center for Neurological Diseases, Jinan, Shandong, P.R. China; d Medical Science and Technology Innovation Center, Shandong First Medical University & Shandong Academy of Medical Sciences, Jinan, Shandong, P.R. China; e Aging Research Center, Department of Neurobiology, Care Sciences and Society, Karolinska Institutet-Stockholm University, Stockholm, Sweden; f Stockholm Gerontology Research Center, Stockholm, Sweden

**Keywords:** Cardiovascular diseases, cognitive function, serum adhesion molecules, ventricular depolarization and repolarization intervals

## Abstract

**Background:**

Emerging evidence has linked electrocardiographic parameters with serum adhesion molecules and cognition; however, their interrelationship has not been explored.

**Objective:**

We sought to investigate the associations of ventricular depolarization and repolarization intervals with serum adhesion molecules and cognitive function among rural-dwelling older adults.

**Methods:**

This population-based study engaged 4,886 dementia-free participants (age ≥60 years, 56.2% women) in the baseline examination (March-September 2018) of MIND-China. Of these, serum intercellular and vascular adhesion molecules (ICAM-1 and VCAM-1) were measured in 1591 persons. We used a neuropsychological test battery to assess cognitive function. Resting heart rate, QT, JT intervals, and QRS duration were assessed with electrocardiogram. Data were analyzed using general linear models adjusting for multiple confounders.

**Results:**

Longer JT interval was significantly associated with lower z-scores of global cognition (multivariable-adjusted β= –0.035; 95% confidence interval = –0.055, –0.015), verbal fluency (–0.035; –0.063, –0.007), attention (–0.037; –0.065, –0.010), and executive function (–0.044; –0.072, –0.015), but not with memory function (–0.023; –0.054, 0.009). There were similar association patterns of QT interval with cognitive functions. In the serum biomarker subsample, longer JT and QT intervals remained significantly associated with poorer executive function and higher serum adhesion molecules. We detected statistical interactions of JT interval with adhesion molecules (p_interaction_ <0.05), such that longer JT interval was significantly associated with a lower executive function z-score only among individuals with higher serum ICAM-1 and VCAM-1.

**Conclusion:**

Longer ventricular depolarization and repolarization intervals are associated with worse cognitive function in older adults and vascular endothelial dysfunction may play a part in the associations.

## INTRODUCTION

Cognitive impairment and cardiac diseases (e.g., arrhythmia and coronary heart disease [CHD]) are highly prevalent among the elderly. Over the past 20 years, evidence has been accumulated that lifelong cardiovascular risk factors and related cardiovascular diseases (CVDs) contribute to accelerated cognitive decline and increased risks of dementia, Alzheimer’s disease (AD), and vascular dementia (VaD) [1]. Therefore, understanding the potential mechanisms linking cardiovascular risk burden with cognitive dysfunction in the aging process is critical to further advance this filed of research. The electrocardiogram (ECG), as a noninvasive, inexpensive, and easily available examination, can monitor complex electrophysiological activity during each cardiac cycle [2]. Specifically, prolonged ventricular depolarization and repolarization intervals are closely related with impaired cardiac blood pumps and are also risk factors for severe arrhythmia and major cardiovascular events (e.g., sudden cardiac arrest, stroke, and CHD) [3–5], which in turn could be linked with poor cognitive function in older adults [6].

However, population-based studies have rarely investigated the relationship between ventricular repolarization and cognitive phenotypes in old age. The cross-sectional data from the Prospective Study of Pravastatin in the Elderly at Risk (PROSPER) showed that a longer QT interval was associated with worse global cognitive performance [7]. By contrast, a small study of biracial community-dwelling older adults found no association between the heart rate-corrected QT (QTc) interval and global cognitive function [8]. Thus, population-based studies are needed to clarify the relationships of ventricular depolarization and repolarization with cognition in the general population of older adults.

Adhesion molecules such as intercellular adhesion molecule 1 (ICAM-1) and vascular cellular adhesion molecule 1 (VCAM-1) can be released into circulatory system when vasculature is damaged, accompanied by vascular inflammation and endothelial dysfunction [9]. Therefore, higher serum concentrations of adhesion molecules are considered biomarkers for clinical, and even subclinical, cardiovascular and cerebrovascular endothelial dysfunction and disorders, which may be linked with cardiac electrophysiological dysfunction and further to cerebral hypoperfusion and ischemic and neurodegenerative lesions [10–14]. Indeed, previous studies have suggested that vascular inflammatory factors are associated with prolonged ventricular repolarization [15, 16], and that high serum adhesion molecules are linked with cerebral microvascular lesions and cognitive decline [17–19]. Taken together, we hypothesized that serum adhesion molecules (e.g., ICAM-1 and VCAM-1), as biomarkers of vascular endothelial dysfunction and disorders, could be associated with both prolonged ventricular repolarization and poor cognitive function in older adults, and that the association of ventricular repolarization with cognitive function might vary by levels of serum adhesion molecules (Supplementary Figure 1).

Thus, in this population-based study, we sought to test this hypothesis by investigating the interrelationships between ventricular depolarization and repolarization intervals, serum adhesion molecules, and cognitive performance among rural-dwelling dementia-free older adults, and further to explore the potential effect modification of vascular adhesion molecules in the associations of ventricular repolarization with cognition.

## METHODS

### Study population

**Fig. 1:**
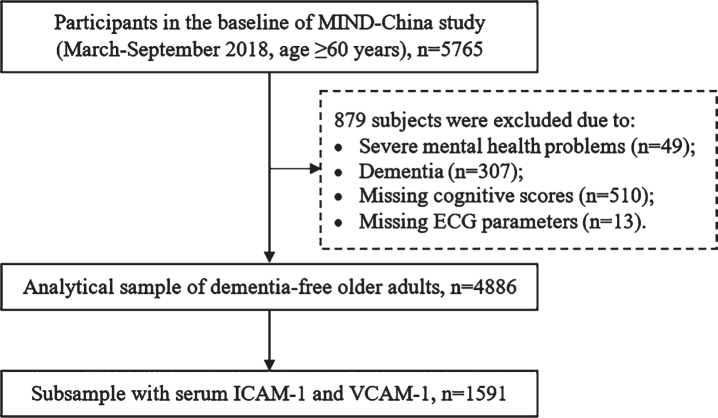
Flowchart of the study participants. MIND-China, the Multimodal INtervention to delay Dementia and disability in the rural China; ECG, electrocardiogram; ICAM-1, intercellular adhesion molecule 1; VCAM-1, vascular cell adhesion molecule 1.

This was a population-based cross-sectional study. Study participants were derived from the ongoing cluster-randomized controlled Multimodal INterventions to delay Dementia and disability in rural China (MIND-China), which is a participating project in the World-Wide FINGERS Network [20, 21]. Briefly, MIND-China targeted people who were aged ≥60 years and living in rural communities (52 villages) of Yanlou Town, Yanggu County, western Shandong Province. In March-September 2018, 5,765 participants took part in the baseline survey of MIND-China, as previously reported [21]. Of these, 879 participants were excluded due to severe mental health problems (*n* = 49), prevalent dementia (*n* = 307), missing cognitive tests (*n* = 510), and missing ECG parameters (*n* = 13), leaving 4,886 participants for the analysis involving ECG parameters in association with cognitive function. Out of these participants, serum adhesion molecules were available in a subsample of 1,591 participants who were randomly selected from the total sample using a cluster (village)-based sampling method (Fig. 1).

The Ethics Committee at Shandong Provincial Hospital reviewed and approved the protocol of MIND-China. Written informed consent was obtained from all the participants, or in the case of cognitively impaired persons, from proxies. Research within the MIND-China project has been conducted in accordance with the ethical principles for medical research involving human subjects expressed in the Declaration of Helsinki. MIND-China was registered in the Chinese Clinical Trial Registry (registration no.: ChiCTR1800017758).

### Data collection and assessments

In March-September 2018, trained medical staff collected data via face-to-face interviews, clinical examinations, cognitive testing, and laboratory tests following a structured questionnaire [21]. Data included demographic features (e.g., age, sex, and education), weight and height, lifestyles (e.g., smoking, alcohol consumption, and leisure-time physical activities), medical history, and current use of medications. Diabetes mellitus, dyslipidemia, and hypertension were defined following the approaches as previously described [22]. Major CVDs, including coronary heart disease, heart failure, arrhythmia, stroke, and transient ischemic attacks, were defined according to self-reported history of the disease or diagnosed by the examining clinicians based on ECG, clinical and neurological examination, or medical history. Arrhythmia included atrial fibrillation and other cardiac conditions that affected the interval of ventricular depolarization and repolarization (e.g., premature atrial and ventricular contractions, second- and third-degree AV-blocks, pacemaker, and supraventricular arrhythmia). Information on the current use of medications was collected during the in-person interviews, and whenever available, drug prescriptions and containers were checked to verify the information. All medications were classified and coded according to the Anatomical Therapeutic Chemical (ATC) classification system, as previously described [23]. According to the previous reports [5], QT-prolonging drugs included amiodarone (ATC code: C01BD01), olanzapine (ATC code: N05AH03), fluphenazine (ATC code: N05AB02), tamoxifen (ATC code: L02BA01), and indapamide (ATC code: C03BA11). DNA was isolated from peripheral blood samples and multiplex polymerase chain reaction was used to determine apolipoprotein E (*APOE*) genotype. *APOE* genotype was dichotomized into carriers versus non-carriers of the ɛ4 allele.

### Assessment of ventricular depolarization and repolarization parameters

A 12-lead ECG was recorded using the three-channel electrocardiograph (CM300, COMEN, Shenzhen, China). Ten-second ECG recordings were completed in a resting, supine position. The resting heart rate, QRS, QT intervals were derived from ECG recordings. The JT interval was defined as the length of the QT interval minus the duration of the QRS complex.

### Assessment of cognitive function

Cognitive function was assessed using a neuropsychological test battery, as previous reported [21, 24]. Briefly, we assessed function of four specific cognitive domains: episodic memory (Auditory Verbal Learning Test [AVLT]-immediate recall, long-delayed free recall, and long-delayed recognition), verbal fluency (Verbal Fluency Test-categories of animals, fruits, and vegetables), attention (Digit Span Test [DST] forward and Trail Making Test [TMT] A), and executive function (DST-backward and TMT B). The raw test scores were standardized into z-scores using the means and standard deviations (SDs), derived from all the baseline participants in MIND-China who were free from dementia. Because all cognitive domains were assessed using more than one test, we created the composite z-score for each of the cognitive domains by averaging the z-scores of the tests for that domain [24]. The global cognitive function was assessed by averaging the z-scores of all four domains.

### Serum adhesion molecules measurement

Peripheral blood samples were drawn into vacutainers with procoagulant separating gel and centrifuged to acquire serum, and then it was aliquoted and stored at –80°C for future analysis, as previously described [21]. Serum ICAM-1 and VCAM-1 concentrations were measured on a Meso Scale Discovery platform (Meso Scale Discovery, Rockville, MD, USA) by V-PLEX Human Vascular Injury Panel following the manufacturer’s instructions [25]. For each plate, one internal control in duplicate was applied and the inter-assay coefficient of variability (CV) and inter-plate inter-assay CV remained within 20%.

### Statistical analysis

Descriptive analysis was performed to report mean (SD) for continuous variables and frequency (percentage) for categorical variables. All ECG parameters and serum adhesion molecules were standardized (mean = 0, SD = 1) prior to the analysis. We compared characteristics of the participants with or without CVDs using chi-squared test for categorical variables and Mann-Whitney U test for continuous variables with non-normal distribution. The associations of the ventricular depolarization and repolarization intervals with cognitive z-scores and serum adhesion molecules were assessed using the multivariable general linear regression models.

We reported the main results from three models. Model 1 was adjusted for age, sex, education, and resting heart rate squared; model 2 was additionally adjusted for body mass index, APOE ɛ4 allele, smoking, alcohol drinking, physical exercise, hypertension, diabetes, dyslipidemia; and in model 3, CVDs and use of QT-prolonging medication variables were added to model 2. We created a dummy variable to indicate a group of individuals with missing values for each of covariates [26].

We tested statistical interactions of ECG parameters with age strata (≥75 versus <75 years), sex, and serum ICAM-1 and VCAM-1 level strata (above median versus below median) on cognitive function. When a statistical interaction was detected (p for interaction <0.05), we further performed the stratified analyses. To assess the impact of CVDs and relevant drug therapy on the associations of ventricular repolarization and serum adhesion molecules with cognitive functions, in the sensitivity analysis we repeated the main analyses by excluding participants with CVDs and use of QT-prolonging medications. The statistical analyses were performed using the Stata Statistical Software: Release 16.0 for Windows (StataCorp LLC., College Station, TX, USA). Two-tailed *p* < 0.05 was considered to be statistically significant.

## RESULTS

### Characteristics of study participants

Of the 5,765 baseline participants, 4,886 (84.75%) were included in the final analytical sample. Compared with people who were excluded, participants included in the analytical sample were younger (mean age 70.04 versus 75.61 years, *p* < 0.001), had a lower proportion of females (56.24% versus 62.46%, *p* < 0.001), and had more years of education (3.41 versus 1.79 years, *p* < 0.001). Table 1 shows the characteristics of study participants in the total sample and by CVDs. The mean age of the 4,886 participants was 70.04 (SD, 5.13) years, 56.24% were female, and 36.53% were illiterate (no formal schooling education). Compared to participants without CVDs, those with prevalent CVDs were older, more likely to be women and have hypertension, diabetes, and dyslipidemia, and less likely to smoke, drink alcohol, and be physically inactive. Participants with CVDs had longer QT interval (*p* < 0.05) and longer QRS duration (*p* < 0.01) than those without. Furthermore, participants with CVDs had a lower z-score in global cognition, memory, and verbal fluency than those without (for all *p* < 0.001).

**Table 1:** Characteristics of the study participants by cardiovascular disease (*n* = 4,886)

Characteristics	Total sample (*n* = 4,886)	Cardiovascular disease		
		No (*n* = 2,902)	Yes (*n* = 1,984)	*p*
Age, y	70.04 (5.13)	69.73 (5.09)	70.50 (5.14)	<0.001
Female, *n* (%)	2,748 (56.24)	1,589 (54.76)	1,159 (58.42)	0.011
Education, y	3.41 (3.53)	3.38 (3.53)	3.46 (3.53)	0.498
Body mass index^a^, kg/m^2^	24.99 (3.75)	24.70 (3.68)	25.42 (3.82)	<0.001
Systolic blood pressure, mmHg	143.77 (21.25)	142.98 (21.36)	144.90 (21.06)	0.001
Diastolic blood pressure, mmHg	84.95 (10.98)	84.77 (10.83)	85.22 (11.18)	0.095
Fasting blood glucose, mmol/L	5.57 (1.40)	5.50 (1.28)	5.68 (1.55)	<0.001
*APOE* ɛ4 allele^a^, *n* (%)	756 (15.86)	437 (15.43)	319 (16.49)	0324
Smoking, *n* (%)				<0.001
Never	3,081 (63.06)	1,805 (62.20)	1,276 (64.31)
Former	732 (14.98)	393 (13.54)	339 (17.09)
Current	1,073 (21.96)	704 (24.26)	369 (18.60)
Alcohol drinking, *n* (%)				<0.001
Never	2,913 (59.62)	1,688 (58.17)	1,225 (61.74)
Former	325 (6.65)	167 (5.75)	158 (7.96)
Current	1,648 (33.73)	1,047 (36.08)	601 (30.29)
Current exercising, *n* (%)	897 (18.36)	486 (16.75)	411 (20.72)	<0.001
Hypertension^a^, *n* (%)	3,247 (66.46)	1,834 (63.2)	1,413 (71.22)	<0.001
Diabetes, *n* (%)	698 (14.29)	350 (12.06)	348 (17.54)	<0.001
Dyslipidemia, *n* (%)	1,169 (23.93)	624 (21.50)	545 (27.47)	<0.001
Stroke, *n* (%)	719 (14.72)	–	719 (39.24)	–
Coronary heart disease, *n* (%)	1,009 (20.65)	–	1,009 (50.86)	–
Heart failure, *n* (%)	131 (2.68)	–	131 (6.60)	–
Transient ischemic attacks, *n* (%)	52 (1.06)	–	52 (2.62)	–
Arrhythmias, *n* (%)	680 (13.92)	–	680 (34.27)	–
Use of QT-prolonging drugs, *n* (%)	51 (1.04)	15 (0.52)	36 (1.81)	<0.001
Antihypertensive drugs, *n* (%)	971 (19.87)	430 (14.82)	541 (27.27)	<0.001
ECG parameters
Heart rate, bpm	67.06 (10.88)	66.5 (9.91)	67.87 (12.12)	0.002
QT interval, ms	397.63 (31.07)	396.76 (29.70)	398.89 (32.94)	0.018
JT interval, ms	299.49 (33.75)	299.3 (33.23)	299.77 (34.49)	0.644
QRS duration, ms	97.57 (12.55)	96.8 (11.33)	98.7 (14.06)	0.003
Cognitive z-scores
Global cognition	–0.03 (0.65)	–0.01 (0.65)	–0.06 (0.64)	0.004
Memory	–0.01 (0.87)	0.01 (0.88)	–0.05 (0.86)	0.003
Verbal fluency	0.01 (0.80)	0.04 (0.80)	–0.03 (0.79)	<0.001
Attention	–0.03 (0.86)	–0.02 (0.86)	–0.06 (0.84)	0.219
Executive function	–0.09 (0.91)	–0.08 (0.92)	–0.10 (0.89)	0.848
Serum adhesion molecule
ICAM-1, ng/ml	478.55 (102.18)	476.52 (98.89)	481.93 (107.41)	0.474
VCAM-1, ng/ml	624.88 (125.69)	621.43 (126.24)	630.61 (124.65)	0.115

*APOE*, apolipoprotein E gene; bpm, beats per minute; ECG, electrocardiograph; ICAM-1, intercellular adhesion molecule 1; VCAM-1, vascular cellular adhesion molecule 1. Data are mean (standard deviation), unless otherwise specified. ^a^Numbers of participants with missing values were 120 for *APOE* genotype, 25 for body mass index, and 41 for hypertension. In subsequent analyses, participants with missing values for each of these variables were replaced with a dummy variable.

Among the ECG parameters, the QT interval was strongly correlated with JT interval (Pearson correlation coefficient *r* = 0.920, *p* < 0.001) and weakly correlated with QRS duration (*r* = 0.148, *p* < 0.001). The JT interval was weakly correlated with QRS duration (*r* = –0.250, *p* < 0.001). Of the 4886 participants in the total analytical sample, data on serum biomarkers were available in 1,591 persons. People in the blood subsample were slightly younger, more likely to be female, and had a higher fasting blood glucose, less likely to have CVDs than those who did not have serum biomarkers (Supplementary Table 1).

### Associations of ventricular depolarization and repolarization intervals with cognitive function (n = 4,886)

Longer QT and JT intervals were significantly associated with lower z-scores of global cognition, verbal fluency, attention, and executive function, but not with memory z-score (Table 2). Specifically, the multivariable-adjusted β-coefficients (95% confidence interval [CI]) of cognitive z-scores associated with JT interval were –0.035 (–0.055, –0.015) for global cognition, –0.035 (–0.063, –0.007) for verbal fluency, –0.037 (–0.065, –0.010) for attention, –0.044 (–0.072, –0.015) for executive function, and –0.023 (–0.054, 0.008) for memory (Table 2, model 3). QRS duration was not significantly associated with z-scores of global cognition and any of the four cognitive domains.

**Table 2:** Associations of ventricular depolarization and repolarization intervals with cognitive function in the total sample (*n* = 4,886)

ECG parameters,	β coefficient (95% confidence interval), cognitive z-score		
per 1-SD increase	Model 1^#^	Model 2^#^	Model 3^#^
Global cognition
QT interval, ms	–0.032 (–0.052, –0.012)^†^	–0.032 (–0.052, –0.012)^†^	–0.032 (–0.053, –0.012)^†^
JT interval, ms	–0.034 (–0.054, –0.014)^†^	–0.035 (–0.055, –0.015)^†^	–0.035 (–0.055, –0.015)^†^
QRS duration, ms	0.004 (–0.012, 0.020)	0.006 (–0.010, 0.021)	0.005 (–0.011, 0.021)
Memory
QT interval, ms	–0.021 (–0.052, 0.010)	–0.021 (–0.052, 0.010)	–0.022 (–0.054, 0.009)
JT interval, ms	–0.022 (–0.053, 0.009)	–0.023 (–0.055, 0.008)	–0.023 (–0.054, 0.008)
QRS duration, ms	0.003 (–0.022, 0.027)	0.004 (–0.020, 0.028)	0.002 (–0.023, 0.027)
Verbal fluency
QT interval, ms	–0.032 (–0.060, –0.004)^*^	–0.031 (–0.059, –0.003)^*^	–0.031 (–0.059, –0.002)^*^
JT interval, ms	–0.035 (–0.063, –0.007)^*^	–0.035 (–0.063, –0.007)^*^	–0.035 (–0.063, –0.007)^*^
QRS duration, ms	0.005 (–0.017, 0.027)	0.007 (–0.014, 0.029)	0.008 (–0.014, 0.030)
Attention
QT interval, ms	–0.037 (–0.064, –0.010)^†^	–0.037 (–0.064, –0.009)^*^	–0.036 (–0.064, –0.009)^*^
JT interval, ms	–0.039 (–0.066, –0.011)^†^	–0.038 (–0.065, –0.011)^*^	–0.037 (–0.065, –0.010)^*^
QRS duration, ms	0.003 (–0.018, 0.025)	0.003 (–0.018, 0.025)	0.003 (–0.019, 0.024)
Executive function
QT interval, ms	–0.039 (–0.067, –0.010)^†^	–0.038 (–0.066, –0.009)^†^	–0.040 (–0.069, –0.012)^†^
JT interval, ms	–0.042 (–0.070, –0.013)^†^	–0.043 (–0.071, –0.014)^†^	–0.044 (–0.072, –0.015)^†^
QRS duration, ms	0.005 (–0.017, 0.027)	0.009 (–0.014, 0.031)	0.007 (–0.016, 0.029)

ECG, electrocardiograph; SD, standard deviation. ^#^Model 1 was adjusted for age, sex, education, and resting heart rate squared; model 2 was additionally adjusted for body mass index, *APOE* ɛ4 allele, smoking, alcohol drinking, physical activity, hypertension, diabetes, and dyslipidemia; in model 3, the presence of prevalent cardiovascular disease (i.e., heart failure, coronary heart disease, arrhythmia, stroke, and transient ischemic attacks) and use of QT-prolonging medications were added to model 2. ^*^p < 0.05, ^†^*p* < 0.01.

### Associations of ventricular depolarization and repolarization parameters with serum adhesion molecules (n = 1,591)

In the subsample of participants with available information on serum adhesion molecules (*n* = 1,591), longer intervals of QT and JT were significantly associated with higher serum ICAM-1, with the multivariable-adjusted β coefficient being 0.059 (95% CI 0.004, 0.113) and 0.089 (0.035, 0.142), respectively, whereas longer QRS duration was significantly associated with lower serum ICAM-1 (multivariable-adjusted β= –0.056; 95% CI = –0.100, –0.012) (Table 3). Similarly, longer QT and JT intervals were significantly associated with higher serum VCAM-1, while longer QRS duration was marginally associated with lower serum VCAM-1 (Table 3). In addition, the associations of longer QT and JT intervals with lower executive function z-score in the subsample remained statistically significant, even in the multivariable-adjusted models (Supplementary Table 2).

**Table 3:** Associations of ventricular depolarization and repolarization intervals with serum adhesion molecules (*n* = 1,591)

ECG parameters,	β coefficient (95% confidence interval), serum adhesion molecules		
per 1-SD increase	Model 1^#^	Model 2^#^	Model 3^#^
ICAM-1, ng/ml
QT interval	0.062 (0.008, 0.116)^*^	0.061 (0.007, 0.115)^*^	0.059 (0.004, 0.113)^*^
JT interval	0.089 (0.035, 0.142)^†^	0.088 (0.034, 0.141)^†^	0.089 (0.035, 0.142)^†^
QRS duration	–0.048 (–0.092, –0.004)^*^	–0.048 (–0.092, –0.004)^*^	–0.056 (–0.100, –0.012)^*^
VCAM-1, ng/ml
QT interval	0.062 (0.008, 0.116)^*^	0.064 (0.009, 0.119)^*^	0.061 (0.005, 0.116)^*^
JT interval	0.088 (0.034, 0.141)^†^	0.087 (0.033, 0.142)^†^	0.085 (0.031, 0.140)^†^
QRS duration	–0.048 (–0.091, –0.004)^*^	–0.044 (–0.089, 0.001)	–0.047 (–0.092, –0.002)^*^

ECG, electrocardiograph; SD, standard deviation; ICAM-1, intercellular adhesion molecule 1; VCAM-1, vascular cellular adhesion molecule 1. ^#^Model 1 was adjusted for age, sex, education, and resting heart rate squared; model 2 was additionally adjusted for body mass index, *APOE* ɛ4 allele, smoking, alcohol drinking, physical activity, hypertension, diabetes, and dyslipidemia; in model 3, the presence of prevalent cardiovascular disease (i.e., heart failure, coronary heart disease, arrhythmia, stroke, and transient ischemic attacks), and use of QT-prolonging medication were added to model 2. ^*^p < 0.05, ^†^*p* < 0.01.

### Interactions of serum adhesion molecules with ventricular repolarization parameters on executive function (n = 1,591)

**Fig. 2:**
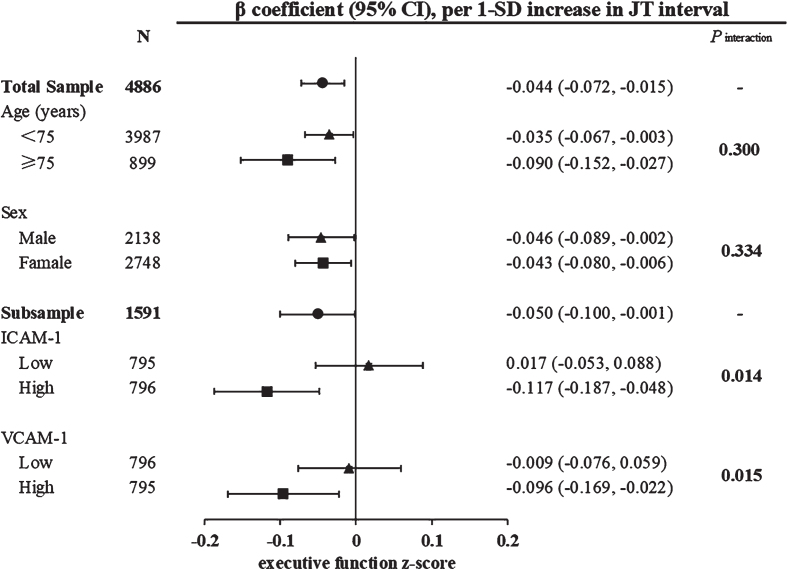
Associations of ventricular repolarization index (JT interval) with executive function by age groups (<75 versus ≥75 years), sex, and serum adhesion molecules. β coefficients (95% confidence intervals) were adjusted for age, sex, education, resting heart rate squared, body mass index, *APOE* 4 allele, current smoking, current alcohol drinking, physical activity, hypertension, diabetes, dyslipidemia, and use of QT prolonged medication. Serum ICAM-1 and VCAM-1 were dichotomized into low and high levels according to their median. CI, confidence interval; SD, standard deviation; ICAM-1, intercellular adhesion molecule 1; VCAM-1, vascular cellular adhesion molecule 1.

Serum ICAM-1 and VCAM-1 concentrations were moderately strong correlated (Pearson correlation coefficient *r* = 0.576, *p* < 0.001). When serum ICAM-1 and VCAM-1 were dichotomized according to their median values (i.e., 469.595 ng/ml for ICAM-1 and 610.326 ng/ml for VCAM-1), we detected a statistical interaction of ventricular repolarization intervals with serum adhesion molecules (low versus above median) on executive function in multivariable-adjusted models (p for interaction <0.05). Further analysis stratified by serum ICAM-1 levels revealed that among persons with higher serum ICAM-1, longer JT interval was significantly associated with lower executive function z-score (multivariable-adjusted β= –0.117; 95% CI = –0.187, –0.048), but not in those with lower serum ICAM-1 (Fig. 2). Similarly, among persons with higher serum VCAM-1, longer JT interval was significantly associated with lower executive function z-score (–0.096; –0.169, –0.022), but not in those with lower serum VCAM-1 (Fig. 2). We detected no statistical interaction of ventricular repolarization intervals with serum adhesion molecules on function of any other cognitive domains.

### Sensitivity analysis

To assess the potential impact of CVDs and use of QT-prolonging medications on the relationships of ventricular de/repolarization parameters with serum adhesion molecules and cognitive function, we repeated the analyses among participants who were free from CVDs and use of QT-prolonging medications. The results were overall comparable with those from the total analytical sample, except that the associations of QRS duration with serum adhesion molecules became statistically marginal or non-significant, possibly owing to reduced statistical power (Supplementary Tables 3 and 4).

## DISCUSSION

In this population-based study of rural older adults, we found that longer QT and JT intervals were associated with poorer cognitive performances, especially poorer executive function, even among individuals without overt CVDs. Furthermore, we revealed that the associations between ventricular repolarization parameter (JT interval) and executive function varied by serum adhesion molecules, such that longer JT interval was associated with poorer executive function mainly only among older adults with higher serum ICAM-1 or VCAM-1 levels. These results suggest that abnormal ventricular depolarization and repolarization are clinical markers for poor cognitive function in dementia-free older adults and that vascular endothelial dysfunction may play a part in the association of long JT interval with poor executive function.

The association of cardiac ventricular repolarization with cognitive function has been rarely evaluated so far in the general population settings. We found that longer QT and JT intervals were associated with lower performance in specific cognitive domains independent of clinical CVDs in the community-dwelling older adults. Similarly, the PROSPER study found that longer QT and JT intervals were cross-sectionally associated with worse executive function independent of cardiovascular risk factors [7]. Moreover, a wider spatial QRS-T angle, a marker of heterogeneity in electrical activity of cardiac ventricles, was also associated with a steeper decline in processing speed and memory performance [27]. However, the PROSPER study targeted individuals who were aged 70–82 years and at high cardiovascular risk, which limits the generalizability of their findings to the general population. A small-scale population-based study of healthy inactive older adults from Canada suggested that higher QTc dispersion, a measure of ventricular repolarization reflecting autonomic cardiovascular dysregulation, was associated with lower global and executive function, indicating that ventricular repolarization may be a marker of cognitive performance prior to the onset of clinical CVDs or cognitive impairment [28]. By contrast, the Honolulu-Asia Aging Study of Japanese-American men found no association of prolonged QT interval in midlife with cognitive decline in late life [29]. Of note, selective survival bias might partly contribute to the lack of association because prolonged QT interval was associated with increased cardiovascular mortality [5]. Additionally, the Chicago Health and Aging Project (age ≥65 years, *n* = 831) showed a tendency of association between high QTc interval and low global cognitive performance in a biracial population [8]. A possible explanation for the lack of significant association could be that the study sample was not large enough to detect the weak-to-moderately strong association, as compared with our present study and the PROSPER study that involved over 4,000 participants. Therefore, more large-scale population-based studies of different ethnics are needed to verify the association between ventricular repolarization and cognitive function in older adults.

To the best of our knowledge, this is the first population-based study that examined the associations of ventricular de/repolarization intervals with serum adhesion molecules in older adults. Previously, the Coronary Artery Risk Development in Young Adults (CARDIA) Study of young- and middle-aged adults suggested that higher circulating ICAM-1 at average ages of 32 and 40 years was associated with lower performance in memory, processing speed, and executive function at average age of 50 years [14]. We found that longer QT and JT intervals were associated with higher serum ICAM-1 and VCAM-1 levels among older adults, whereas longer QRS duration was associated with lower serum adhesion molecules. These studies suggest that endothelial dysfunction is associated with poor cognitive function. We further revealed that the relationship of longer JT interval with poorer executive function appeared to be evident mainly among older adults with high serum adhesion molecules. There are several potential explanations for their associations. First, higher serum ICAM-1 and VCAM-1 levels were associated with coronary atherosclerosis [30–33], which could lead to prolonged ventricular repolarization interval [34, 35]. Second, elevated levels of serum adhesion molecules were correlated with inflammatory biomarkers, which inhibited the transient outward K^+^ current and prolonged ventricular repolarization [15, 16]. Taken together, these studies support the view that high serum ICAM-1 and VCAM-1, as biomarkers of endothelial dysfunction and vascular damage, are associated with ventricular de/repolarization [9, 32].

Several potential pathways may explain the associations of prolonged ventricular depolarization and repolarization intervals with poor cognitive function as well as the effect modification of high serum adhesion molecules. First, prolonged ventricular repolarization intervals and poor cognitive function were correlated as a result of age-related deteriorations in the autonomic function and brain pathology [7, 36]. Indeed, higher levels of serum adhesion molecules may reflect worse cardiovascular and cerebrovascular endothelial function, which may impair both the autonomic nervous system and cognitive function [31, 36]. Second, prolonged ventricular depolarization and repolarization intervals and cognitive impairment share common pathological basis of vascular damage, subclinical atherosclerosis, and clinical CVDs [37]. Third, coronary atherosclerosis promotes the release of cellular adhesion molecules from endothelial cells, while the subsequent coronary slow flow phenomenon may lead to prolonged ventricular repolarization [35, 37]. Then, the prolonged QT interval causes poor left ventricular function and low left ventricular ejection fraction [12], resulting in decreased cardiac output and cerebral blood flow and ischemic brain lesions [13], which may subsequently induce poor performance in executive function [38]. Moreover, cerebrovascular endothelial dysfunction, reflected by higher serum adhesion molecules, may aggravate cerebral hypoperfusion and cognitive impairment [17].

The main strengths of the current study refer to the population-based design of a large sample that targeted the largely ignored demographic group of rural-dwelling Chinese older adults in research on cardiovascular health and cognitive aging as well as adjustment for a range of potential confounding factors. Furthermore, we were able to explore the potential role of serum adhesion molecules in the associations of ventricular de/repolarization parameters with cognitive outcomes in a subsample where epidemiological, cognitive, and clinical data were integrated with serum biomarkers. However, our study does have several limitations. First, the ventricular parameters were assessed by ECG machine, and arrhythmias could affect the accuracy of ECG parameters. However, such an effect was likely to be minimal because we controlled for arrhythmia conditions in the analysis and the main results were also verified in the sensitivity analysis among participants free of clinical CVDs and no use of QT-prolonging drugs. Second, findings from a cross-sectional study do not necessarily indicate any causal relationships and the observed cross-sectional associations may be subject to selective survival bias, which usually leads to an underestimation of the true association. Finally, our study sample was derived from a less developed rural area in China, where a considerable proportion of people had no or limited education and low socioeconomic status. This should be kept in mind when generalizing our findings to other populations.

### Conclusion

In summary, this population-based study found that longer ventricular depolarization and repolarization intervals were associated with worse cognitive function among rural-dwelling older adults independent of overt CVDs. Furthermore, the association of ventricular repolarization parameters with executive function varies by serum concentrations of adhesion molecules. Future prospective cohort studies could help clarify the potential causal relationships of ECG parameters and vascular adhesion molecules with cognitive outcomes and better understand the mechanisms underlying the association between ventricular depolarization and repolarization intervals and cognitive function in old age.

## Supplementary Material

Supplementary Material

## Data Availability

The datasets used and/or analyzed during the current study are available from the corresponding author upon reasonable request and approval by the Steering Committee of MIND-China at the Department of Neurology, Shandong Provincial Hospital.
