# Supplementary material for: Associations of Cardiac Ventricular Repolarization with Serum Adhesion Molecules and Cognitive Function in Older Adults: The MIND-China Study
Source: J Alzheimers Dis. 2023 Mar 7;92(1):273–83. doi: 10.3233/JAD-220874 (PMC10200176; doi:10.3233/JAD-220874)
Supplement: Supplementary Material [file jad-92-jad220874-s001.pdf]

Supplementary Material

Associations of Cardiac Ventricular Repolarization with Serum Adhesion Molecules and Cognitive Function in Older Adults: The MIND-China Study

Supplementary Table 1. Characteristics of participants included and excluded in the analysis of biomarker subsample

Characteristics	Total sample	Serum adhesion molecules		
	(n=4,886)	No (n=3,295)	Yes (n=1,591)	p
Age, y	70.04 (5.13)	70.28 (5.35)	69.54 (4.59)	0.003
Female, n (%)	2,748 (56.24)	1,817 (55.14)	931 (58.52)	0.026
Education, y	3.41 (3.53)	3.40 (3.53)	3.44 (3.52)	0.498
BMI, kg/m^2	24.99 (3.75)	24.95 (3.84)	25.09 (3.57)	0.116
SBP, mmHg	143.76 (21.25)	143.69 (21.36)	143.92 (21.05)	0.574
DBP, mmHg	84.95 (10.98)	84.95 (11.11)	84.97 (10.69)	0.808
FPG, mmol/L	5.57 (1.40)	5.57 (1.45)	5.58 (1.29)	0.009
APOE ε4 allele, n (%)	756 (15.86)	524 (16.41)	232 (14.76)	0.143
Smoking, n (%)				0.128
Never	3,081 (63.06)	2,050 (62.22)	1,031 (64.80)	
Former	732 (14.98)	495 (15.02)	237 (14.90)	
Current	1,073 (21.96)	750 (22.76)	323 (20.30)	
Alcohol drinking, n (%)		1,283 (31.01)	540 (33.29)	0.121
Never	2,913 (59.62)	1,944 (59.00)	969 (60.91)	
Former	325 (6.65)	235 (7.13)	90 (5.66)	
Current	1,648 (33.73)	1,116 (33.87)	532 (33.44)	
Current exercising, n (%)	897 (18.36)	542 (16.45)	355 (22.31)	<0.001
Hypertension, n (%)	3,247 (66.46)	2,162 (65.61)	1,085 (68.20)	0.073
Diabetes, n (%)	698 (14.29)	466 (14.14)	232 (14.58)	0.681
Dyslipidemia, n (%)	1,169 (23.93)	796 (24.16)	373 (23.44)	0.584
Stroke, n (%)	719 (14.72)	502 (15.24)	217 (13.64)	0.140
CHD, n (%)	1,009 (20.65)	700 (21.24)	309 (19.42)	0.021
Heart failure, n (%)	131 (2.68)	104 (3.16)	27 (1.70)	0.003
TIA, n (%)	52 (1.06)	46 (1.40)	6 (0.38)	0.001
Arrhythmia, n (%)	680 (13.92)	476 (14.45)	204 (12.82)	0.124
Use of QT-prolonging medications, n (%)	51 (1.04)	37 (1.12)	14 (0.88)	0.234
Use of antihypertensive medications, n (%)	971 (19.87)	629 (19.09)	342 (21.50)	0.048
ECG parameters				
Heart rate, bpm	67.06 (10.88)	67.43 (11.17)	66.28 (10.21)	0.001

QT interval, ms	397.63 (31.07)	397.38 (31.49)	398.14 (30.19)	0.366
JT interval, ms	299.49 (33.75)	299.20 (33.64)	300.09 (33.98)	0.466
QRS duration, ms	97.57 (12.55)	97.64 (12.68)	97.42 (12.28)	0.731
Cognitive z-score				
Global cognition	-0.03 (0.65)	-0.04 (0.63)	-0.02 (0.68)	0.577
Memory	-0.01 (0.87)	-0.01 (0.85)	-0.01 (0.92)	0.459
Language	0.01 (0.80)	0.00 (0.78)	0.04 (0.82)	0.181
Attention	-0.03 (0.86)	-0.05 (0.85)	0.00 (0.86)	0.150
Executive function	-0.09 (0.91)	-0.08 (0.90)	-0.11 (0.93)	0.298

Data are mean (standard deviation), unless otherwise specified.

APOE, Apolipoprotein E gene; bpm, beats per minute; BMI, body mass index; CHD, coronary heart disease; DBP, diastolic blood pressure; ECG, electrocardiograph; FBG, fasting blood glucose; SBP, systolic blood pressure; TIA, transient ischemic attack.

Supplementary Table 2. Associations of ventricular depolarization and repolarization intervals with cognitive performance in the subsample of serum adhesion molecules (n=1591)

ECG parameters, per 1-SD increase	β coefficient (95% confidence interval), cognitive z-score		
	Model 1[#]	Model 2[#]	Model 3[#]
Global cognition			
QT interval, ms	-0.042 (-0.078, -0.006)*	-0.041 (-0.077, -0.005)*	-0.042 (-0.078, -0.005)*
JT interval, ms	-0.042 (-0.077, -0.006)*	-0.041 (-0.077, -0.005)*	-0.040 (-0.075, -0.004)*
QRS duration, ms	0.001 (-0.028, 0.031)	0.003 (-0.027, 0.032)	0.000 (-0.030, 0.029)
Memory			
QT interval, ms	-0.043 (-0.100, 0.014)	-0.043 (-0.100, 0.014)	-0.048 (-0.105, 0.010)
JT interval, ms	-0.027 (-0.083, 0.030)	-0.027 (-0.084, 0.029)	-0.027 (-0.084, 0.030)
QRS duration, ms	-0.024 (-0.071, 0.022)	-0.024 (-0.070, 0.023)	-0.031 (-0.078, 0.015)
Verbal fluency			
QT interval, ms	-0.035 (-0.084, 0.015)	-0.033 (-0.083, 0.016)	-0.031 (-0.081, 0.019)
JT interval, ms	-0.039 (-0.088, 0.011)	-0.038 (-0.087, 0.011)	-0.036 (-0.085, 0.014)
QRS duration, ms	0.008 (-0.032, 0.049)	0.010 (-0.030, 0.050)	0.010 (-0.031, 0.050)
Attention			
QT interval, ms	-0.042 (-0.089, 0.005)	-0.039 (-0.086, 0.008)	-0.038 (-0.085, 0.010)
JT interval, ms	-0.049 (-0.095, -0.003)*	-0.047 (-0.094, -0.001)*	-0.046 (-0.092, 0.001)
QRS duration, ms	0.015 (-0.023, 0.053)	0.016 (-0.022, 0.054)	0.017 (-0.022, 0.055)
Executive function			
QT interval, ms	-0.050 (-0.100, -0.005)*	-0.049 (-0.098, 0.001)	-0.050 (-0.100, 0.000)
JT interval, ms	-0.052 (-0.101, -0.003)*	-0.052 (-0.101, -0.002)*	-0.050 (-0.100, -0.001)*
QRS duration, ms	0.006 (-0.035, 0.046)	0.008 (-0.033, 0.048)	0.004 (-0.037, 0.045)

ECG, electrocardiograph; SD, standard deviation.

[#]Model 1 was adjusted for age, sex, education, and resting heart rate squared; model 2 was additionally adjusted for body mass index, *APOE* ε4 allele, current smoking, alcohol drinking, physical activity, hypertension, diabetes, and dyslipidemia; in model 3, the presence of cardiovascular disease (i.e., heart failure, coronary heart disease, arrhythmia, stroke, and transient ischemic attacks), and use of QT-prolonging medication were added to model 2. *$p<0.05$, †$p<0.01$, ‡$p<0.001$.

Supplementary Table 3. Associations of ventricular depolarization and repolarization intervals with cognitive performance in participants free of CVDs and no use of QT-prolonging medications (n=2,887)

**ECG parameters, per 1-SD increase**	**β coefficient (95% confidence interval), cognitive z-score**	
	**Model 1#**	**Model 2#**
Global cognition		
QT interval, ms	-0.055 (-0.082, -0.027)‡	-0.051 (-0.079, -0.024)‡
JT interval, ms	-0.044 (-0.070, -0.017)†	-0.041 (-0.067, -0.015)†
QRS duration, ms	-0.012 (-0.034, 0.011)	-0.011 (-0.033, 0.012)
Memory		
QT interval, ms	-0.044 (-0.086, -0.001)	-0.041 (-0.084, 0.002)
JT interval, ms	-0.034 (-0.075, 0.008)	-0.031 (-0.073, 0.010)
QRS duration, ms	-0.012 (-0.047, 0.024)	-0.012 (-0.048, 0.023)
Verbal fluency		
QT interval, ms	-0.054 (-0.093, -0.016)*	-0.048 (-0.087, -0.010)*
JT interval, ms	-0.045 (-0.082, -0.008)*	-0.042 (-0.079, -0.004)*
QRS duration, ms	-0.009 (-0.041, 0.023)	-0.005 (-0.037, 0.027)
Attention		
QT interval, ms	-0.049 (-0.087, -0.012)*	-0.048 (-0.085, -0.010)*
JT interval, ms	-0.044 (-0.081, -0.008)*	-0.042 (-0.078, -0.006)*
QRS duration, ms	0.001 (-0.032, 0.030)	-0.003 (-0.034, 0.028)
Executive function		
QT interval, ms	-0.072 (-0.111, -0.033)†	-0.068 (-0.107, -0.029)†
JT interval, ms	-0.052 (-0.090, -0.014)*	-0.049 (-0.087, -0.012)*
QRS duration, ms	-0.026 (-0.058, 0.007)	-0.023 (-0.055, 0.010)

CVD, cardiovascular disease; ECG, electrocardiograph; SD, standard deviation.

#Model 1 was adjusted for age, sex, education, and resting heart rate squared; model 2 was additionally adjusted for body mass index, *APOE* ε4 allele, current smoking, alcohol drinking, physical activity, hypertension, diabetes, and dyslipidemia.

*p<0.05, †p<0.01, ‡p<0.001.

Supplementary Table 4. Associations of ventricular depolarization and repolarization intervals with serum adhesion molecules in participants free of CVDs and no use of QT-prolonging medications (n=989)

ECG parameters, per 1-SD increase	β coefficient (95% confidence interval), serum adhesion molecules	
	Model 1[#]	Model 2[#]
ICAM-1		
QT interval	0.106 (0.034, 0.178)[†]	0.109 (0.037, 0.182)[†]
JT interval	0.131 (0.062, 0.200)[‡]	0.127 (0.058, 0.197)[‡]
QRS duration	-0.060 (-0.117, -0.002)[*]	-0.049 (-0.107, 0.009)
VCAM-1		
QT interval	0.099 (0.023, 0.175)[*]	0.100 (0.023, 0.176)[†]
JT interval	0.121 (0.048, 0.194)[†]	0.118 (0.045, 0.192)[†]
QRS duration	-0.053 (-0.114, 0.006)	-0.049 (-0.110, 0.012)

ICAM-1, intercellular adhesion molecule 1; VCAM-1, vascular cellular adhesion molecule 1; CVD, cardiovascular disease.

[#]Model 1 was adjusted for age, sex, education, and resting heart rate squared; model 2 was additionally adjusted for body mass index, *APOE* ε4 allele, current smoking, alcohol drinking, physical activity, hypertension, diabetes, and dyslipidemia; in model 3, use of QT-prolonging medication were added to model 2.

[*]p<0.05, [†]p<0.01, [‡]p<0.001.

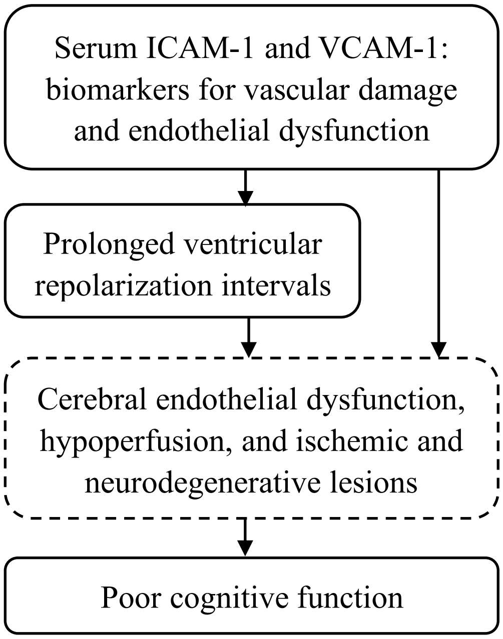

Supplementary Figure 1. Hypothetical model linking serum adhesion molecules and ventricular repolarization intervals with poor cognitive function. Note: High serum adhesion molecules are considered biomarkers for clinical and subclinical cardiovascular and cerebrovascular endothelial dysfunction and disorders, which may be linked with prolonged ventricular repolarization intervals. The prolonged ventricular repolarization intervals can be linked with cerebral hypoperfusion and ischemic and neurodegenerative lesions, which are well known to be associated with poor cognitive function. Pathways in solid-line boxes were examined in this study and those in dashed-line boxes were hypothetical and not directly examined. ICAM-1, intercellular adhesion molecule 1; VCAM-1, vascular cell adhesion molecule 1.
